# Effects of Probiotic Supplement in Pregnant Women with Gestational Diabetes Mellitus: A Systematic Review and Meta-Analysis of Randomized Controlled Trials

**DOI:** 10.1155/2019/5364730

**Published:** 2019-09-05

**Authors:** Jiayue Zhang, Shujuan Ma, Shilan Wu, Chuhao Guo, Sisi Long, Hongzhuan Tan

**Affiliations:** Department of Epidemiology and Biostatistics, Xiangya School of Public Health, Central South University, Changsha, Hunan, China

## Abstract

**Background:**

Previous studies showed that probiotics could improve glycemic control and attenuate some of the adverse effects of type 2 diabetes. However, whether the effects are generalizable to gestational diabetes mellitus (GDM) remains uncertain.

**Objective:**

We conducted a systematic review and meta-analysis to evaluate the effects of probiotic supplement in GDM.

**Method:**

PubMed, EMBASE, the Cochrane Library, and EBSCO were systematically searched for relevant literature published through January 2019. Randomized controlled trials (RCTs) assessing the effects of probiotic supplement on one or more of the following in GDM were included: pregnancy outcome (the primary outcome), glycemic control, blood lipid profile, and inflammation and oxidative stress. Two reviewers independently extracted data and assessed the risk of bias in studies. Meta-analysis was conducted by using the fixed effects model unless substantial heterogeneity was found among studies.

**Results:**

Eleven randomized trials involving 719 participants were included for analysis. Eight of the trials were from Iran. Probiotics were given alone in eight trials and synbiotics in three trials. Though the components of probiotics varied, Lactobacillus was included in all trials and Bifidobacterium in all except one. The duration of intervention ranged from 4 to 8 weeks. Almost all trials (10/11) had a low risk of bias. Probiotic supplementation reduced the risk of a newborn's hyperbilirubinemia by 74% and improved four biomarkers for glycemic control (fasting blood glucose, fasting serum insulin, homeostasis model assessment insulin resistance, and quantitative insulin sensitivity check index), two biomarkers for lipid profile (triglycerides and HDL-cholesterol), and four biomarkers for inflammation and oxidative stress (total glutathione, malondialdehyde, nitric oxide, and total antioxidant capacity). But significant heterogeneity was observed in the meta-analyses on the four biomarkers related to glycemic control and on triglycerides, which could not be explained by prespecified subgroup analyses according to the mean age of participants and intervention type (i.e., probiotics or synbiotics). The effects on the risk of preterm delivery, macrosomia and a newborns' hypoglycemia, gestational age, total cholesterol, and LDL-cholesterol were not statistically significant.

**Conclusion:**

Probiotic supplementation seemed to be able to reduce the risk of a newborn's hyperbilirubinemia and improve glycemic control, blood lipid profiles and inflammation and oxidative stress in pregnant women with GDM. However, due to the heterogeneity among existing studies, the surrogate nature of outcomes, and/or the fact that most studies were from Iran, the clinical significance and generalizability of the above findings remain uncertain. Further studies are warranted to address the limitations of existing evidence and better inform the management of GDM.

## 1. Introduction

Gestational diabetes mellitus (GDM) is defined as any degree of glucose intolerance that occurs or is first recognized during pregnancy [1, 2]. GDM was reported to be associated with various obstetric complications, such as hydramnios, preterm delivery, and cesarean delivery [3], and adverse outcomes of fetuses or newborns, such as congenital malformation, fetal death [4], and neonatal respiratory distress syndrome [2, 5]. In addition, both mothers with GDM and their infants were at an increased risk of diabetes mellitus and metabolic dysfunction in later life [6].

Probiotics was defined by the Food and Agriculture Organization/World Health Organization as “live microorganisms which when administered in adequate amounts confer a health benefit on the host” [7–9]. Probiotics may reinoculate or balance the host's gut microbiota, which are associated with diabetes and other metabolic diseases [10–12]. They can be given as biological supplements or in food such as yogurt [13–15], making them readily available for consumption [16–18].

Previous studies showed that probiotics could improve glycemic control [9, 19–22] and attenuate some of the adverse effects of type 2 diabetes [23]. However, whether the effects are generalizable to GDM remains uncertain. For example, Babadi et al. [24] found that probiotic supplementation could improve fasting blood glucose in pregnant women with GDM, while Ahmadi et al. [25] found that there was no statistically significant difference between probiotics and placebo in glycemic control. Thus, we conducted a systematic review to summarize all available trials and provide a full picture of the effects of probiotics on both hard and surrogate outcomes.

## 2. Materials and Methods

This review was conducted in accordance with the PRISMA statement [26].

### 2.1. Eligibility Criteria

Randomized controlled trials that allocated pregnant women with GDM to an intervention group receiving probiotic supplements or to a control group receiving placebo and reported at least one of the following outcomes were included: (1) pregnancy outcomes (the primary outcome of this systematic review)—preterm birth, macrosomia, gestational age, newborns' hyperbilirubinemia, and newborns' hypoglycemia; (2) blood glucose and related indicators—fasting blood glucose (FBG), fasting serum insulin (FSI), homeostasis model assessment insulin resistance (HOMA-IR), and quantitative insulin sensitivity check index (QUICKI); (3) blood lipid profiles—triglycerides, total cholesterol, LDL-cholesterol, and HDL-cholesterol; and (4) biomarkers of inflammation and oxidative stress—total glutathione (GSH), malondialdehyde (MDA), nitric oxide (NO), and total antioxidant capacity (TAC). To be eligible, probiotics could be given alone or together with prebiotics, which are nondigestible carbohydrates that nourish probiotics and healthy bacteria. The combination of probiotics and prebiotics is usually referred to as synbiotic.

### 2.2. Literature Search and Study Selection

PubMed, Cochrane, EBSCO, and EMBASE were searched for relevant literature published through January 2019 with the following terms: (probiotic∗ OR synbiotic∗ OR lactobacill∗ OR streptococc∗ OR bifidobacter∗ OR saccharomy∗ OR yeast OR yogurt OR bacteria∗ OR acidophilus OR ferment∗ OR microorganism∗) AND (pregnan∗ OR gestation∗ OR matern∗ OR obstetric∗ OR expectan∗) AND (random∗ OR trial∗ OR placebo OR blind∗) AND (diabetes OR glucose). Two reviewers screened titles and abstracts of the retrieved records to select potentially eligible studies, for which full texts were obtained and examined to determine their eligibility. The reference lists of eligible studies and relevant reviews were manually checked for additional studies. Duplicate publications were excluded.

### 2.3. Data Extraction and Quality Assessment

The following data were extracted from eligible studies: first author, year of publication, country of study, number of trial participants, mean age of participants, details of intervention (e.g., probiotic species and probiotic counts measured by colony-forming unit), intervention duration, and main results on the interested outcomes. Data extraction was conducted independently by two reviewers. Disagreements between the two were resolved by discussion until a consensus was achieved. The methodological quality of the included trials was assessed by using the Cochrane Risk of Bias tool for Quality Assessment of Randomized Controlled Trials [27]. This tool rates six domains of primary research, i.e., random sequence generation, allocation concealment, blinding of participants and personnel, blinding of outcome assessment, incomplete outcome data, and selective reporting. If a study had four or more domains (including the “random sequence generation” domain or the “allocation concealment” domain) at a low risk of bias and none at a high risk, the study as a whole would be rated as having low risk bias [16].

### 2.4. Statistical Analyses

RevMan 5.3 software was used for conducting meta-analysis. For binary outcomes, i.e., preterm delivery, macrosomia, newborns' hyperbilirubinemia, and newborns' hypoglycemia, risk ratios (RRs) with 95% confidence intervals (CIs) were combined across relevant studies. For the other outcomes which are all continuous, the differences in prepost changes between the probiotic and placebo groups were combined. The fixed effects model was used for meta-analysis, unless substantial heterogeneity was found among studies. A *P* value ≤ 0.10 for Cochran's *Q* test or an *I*^2^ ≥ 50% was suggestive of substantial heterogeneity, in which case subgroup analyses according to the mean age of participants and the type (probiotic or synbiotic), duration, and dose of intervention were conducted to explore the potential sources. Sensitivity analyses were conducted by excluding the studies with high risk bias to examine the robustness of results. Potential publication bias was assessed using a funnel plot if 10 or more studies were included in a meta-analysis [28]. A two-tailed *P* < 0.05 was considered statistically significant for all analyses except heterogeneity tests.

## 3. Results

**Figure 1:**
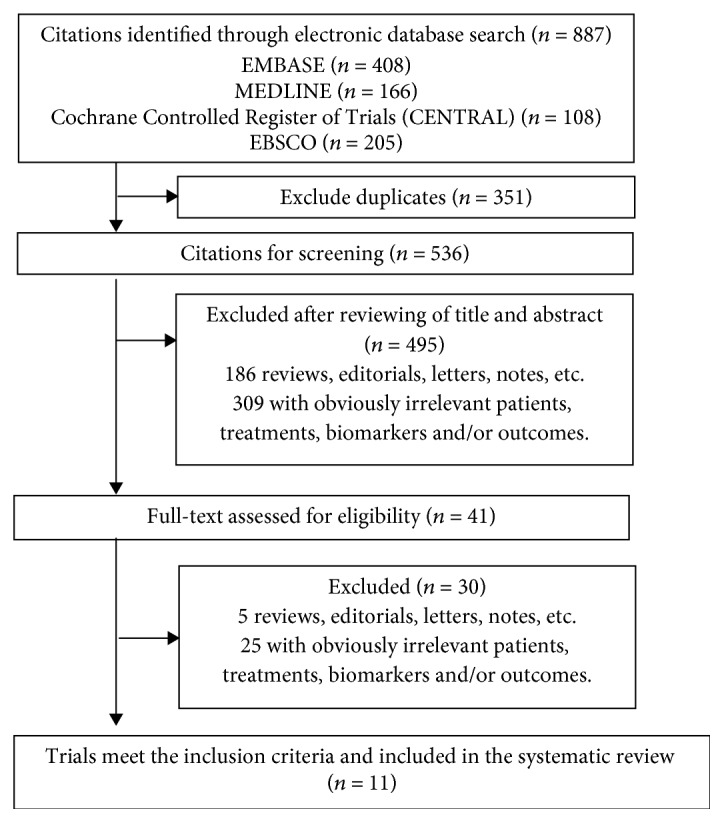
Flowchart of literature selection.

A total of 887 citations were identified by literature search, and 11 randomized trials [24, 25, 29–37] involving 719 participants were finally included in this systematic review (Figure 1). Characteristics of the included studies are shown in Table 1. Eight trials were from Iran, while the other three were from Ireland, Turkey, and Thailand, respectively. The mean age of participants ranged from 26.2 to 33.5 and was above 30 years in 5 studies. Probiotics were given alone in eight trials and synbiotics in three trials. The composition of probiotics varied between studies, but all trials included Lactobacillus, and all except one trial included Bifidobacterium. The duration of intervention ranged from 4 to 8 weeks.

**Figure 2:**
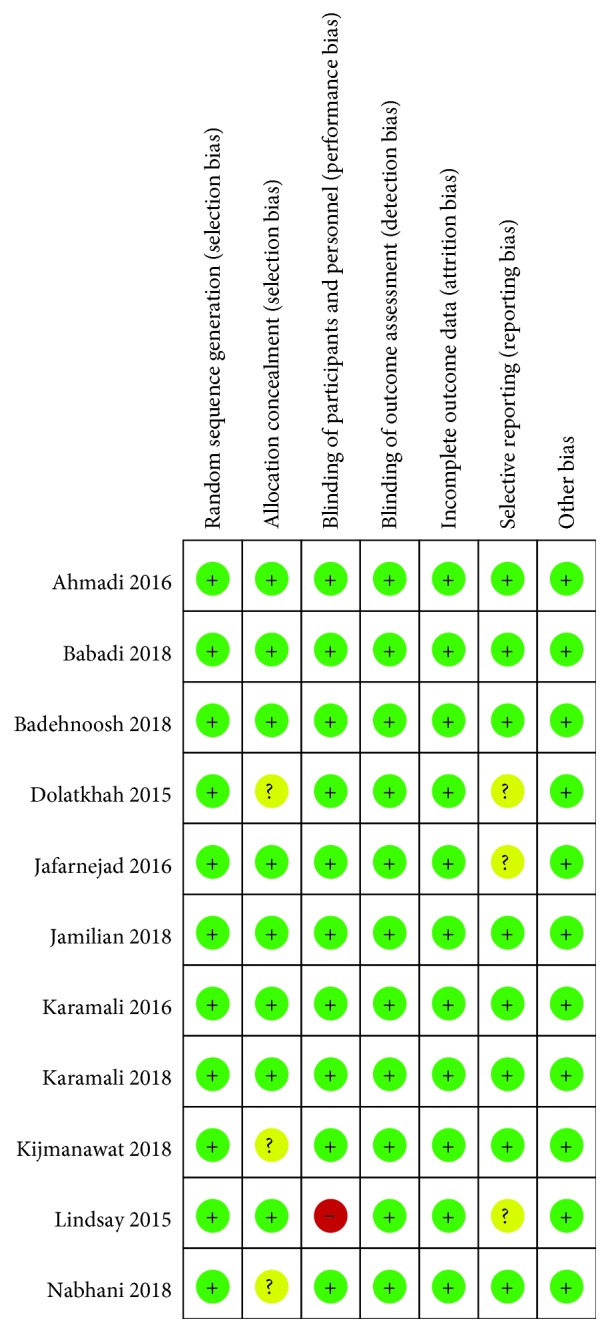
Risk of bias among included randomized controlled trials.

All except one study were considered having a low risk of bias, as assessed using the Cochrane Collaboration Risk of Bias tool (Figure 2). The study by Lindsay et al. was considered having a high risk because the number of participants available for analysis of some outcomes was much smaller than originally randomized.

**Figure 3:**
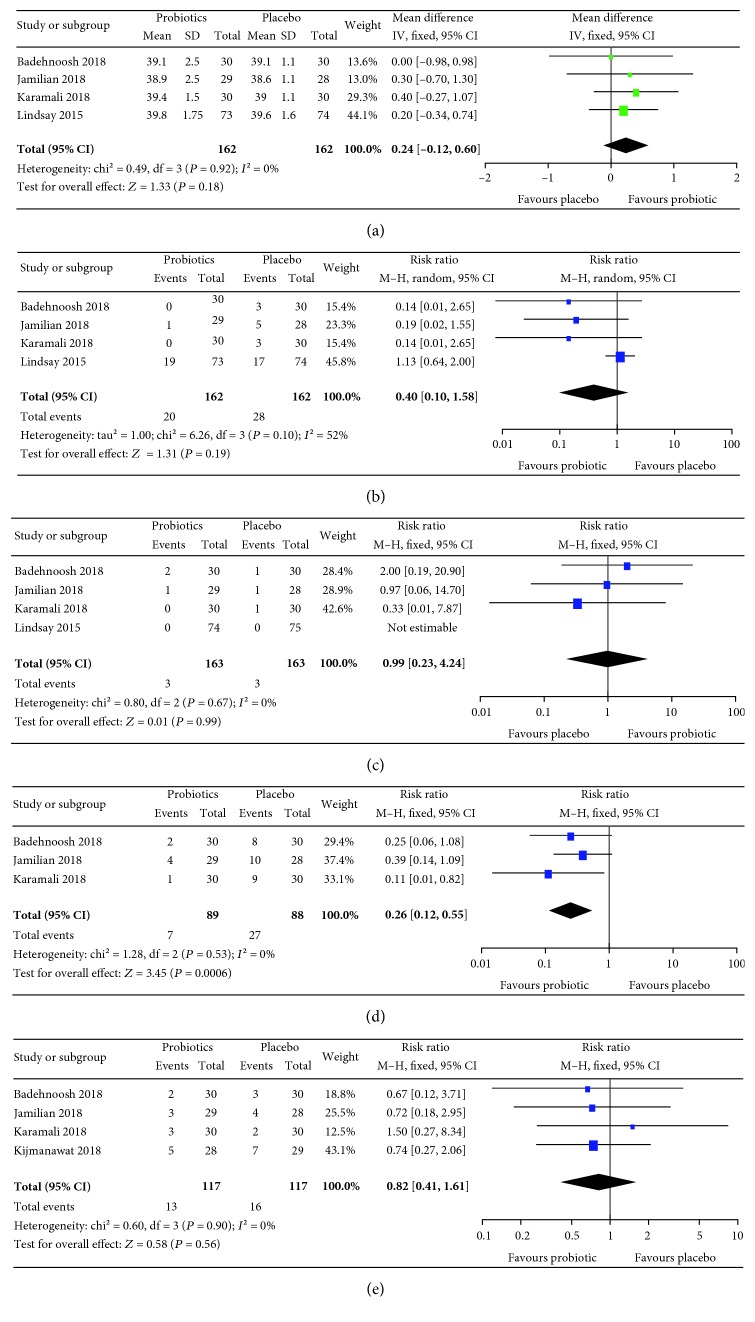
Effect of probiotic supplementation on pregnancy outcomes: (a) gestational age (weeks), (b) the incidence of macrosomia, (c) the incidence of preterm delivery, (d) the incidence of newborns' hyperbilirubinemia, and (e) the incidence of newborns' hypoglycemia in pregnant women with gestational diabetes.

**Figure 4:**
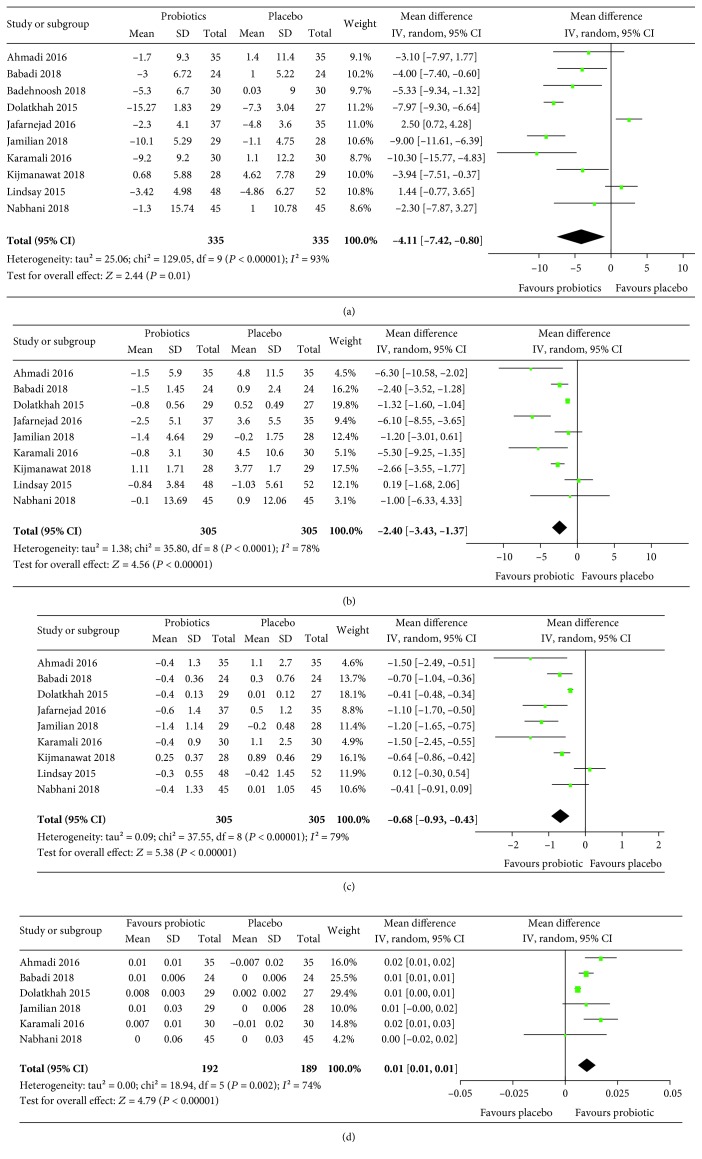
Effect of probiotic supplementation on blood glucose and related indicators: (a) FBG (mg/dL), (b) FSI (*μ*IU/mL), (c) HOMA-IR, and (d) QUICKI in pregnant women with gestational diabetes.

**Figure 5:**
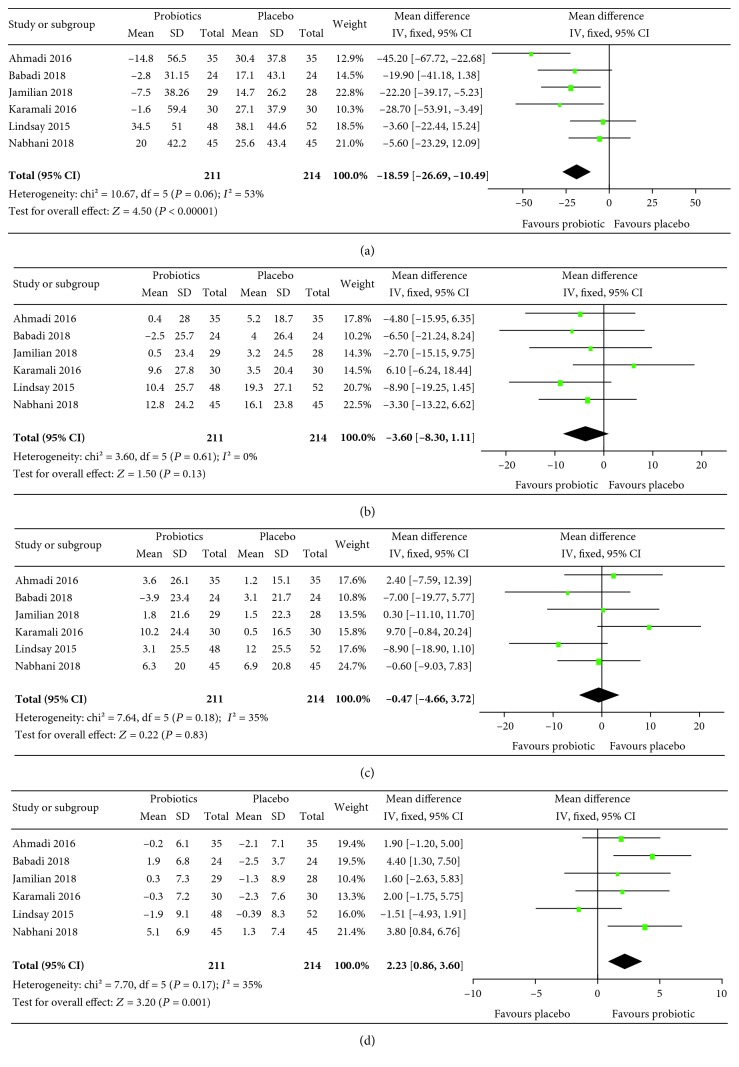
Effect of probiotic supplementation on blood lipid profiles: (a) triglycerides (mg/dL), (b) total cholesterol (mg/dL), (c) LDL-cholesterol (mg/dl), and (d) HDL-cholesterol (mg/dL) in pregnant women with gestational diabetes.

**Figure 6:**
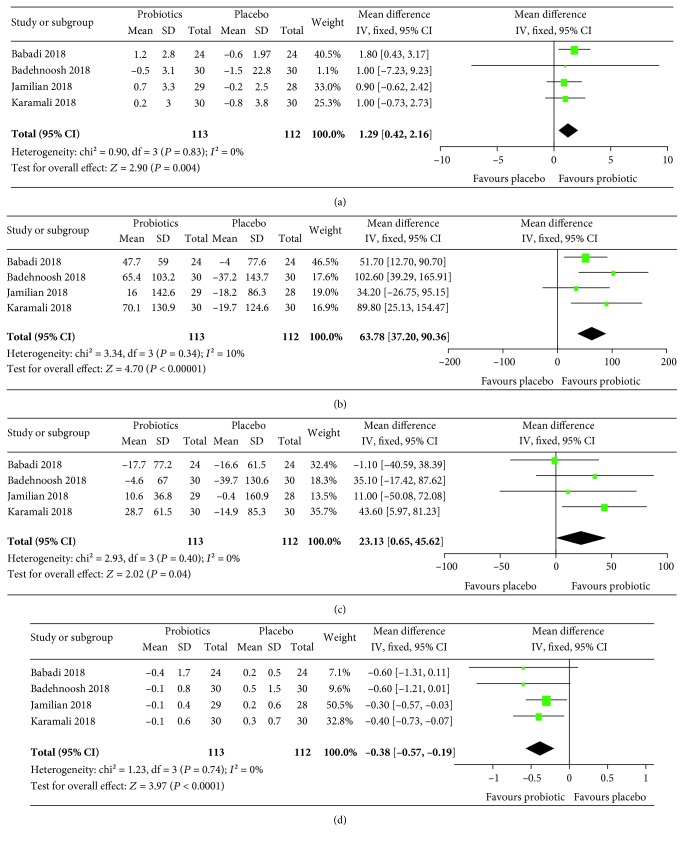
Effect of probiotic supplementation on biomarkers of inflammation and oxidative stress: (a) NO (*μ*mol/L), (b) TAC (mmol/L), (c) GSH (*μ*mol/L), and (d) MDA (*μ*mol/L) in pregnant women with gestational diabetes.

The results of meta-analyses are summarized in Figures 3–6. For pregnancy outcomes, probiotic supplementation reduced the incidence of a newborn's hyperbilirubinemia by 74% (RR: 0.26, 95% CI: 0.12, 0.55), whereas no statistically significant difference was observed in other outcomes between the probiotic and placebo groups. For secondary outcomes, probiotic supplementation improved FBG (mean difference: -4.11 mg/dL, 95% CI: -7.42, -0.80), FSI (mean difference: -2.40 *μ*IU/mL, 95% CI: -3.43, -1.37), HOMA-IR (mean difference: -0.68, 95% CI: -0.93, -0.43), QUICKI (mean difference: 0.01, 95% CI: 0.01, 0.01), triglycerides (mean difference: -18.59 mg/dL, 95% CI: -26.69, -10.49), HDL-cholesterol (mean difference: 2.23 mg/dL, 95% CI: 0.86, 3.60), NO (mean difference: 1.29 *μ*mol/L, 95% CI: 0.42, 2.16), TAC (mean difference: 63.78 mmol/L, 95% CI: 37.20, 90.36), GSH (mean difference: 23.13 *μ*mol/L, 95% CI: 0.65, 45.62), and MDA (mean difference: -0.38 *μ*mol/L, 95% CI: -0.57, -0.19) but had no effects on total cholesterol and LDL-cholesterol.

### 3.1. Subgroup, Sensitivity, and Publication Bias Analysis

**Figure 7:**
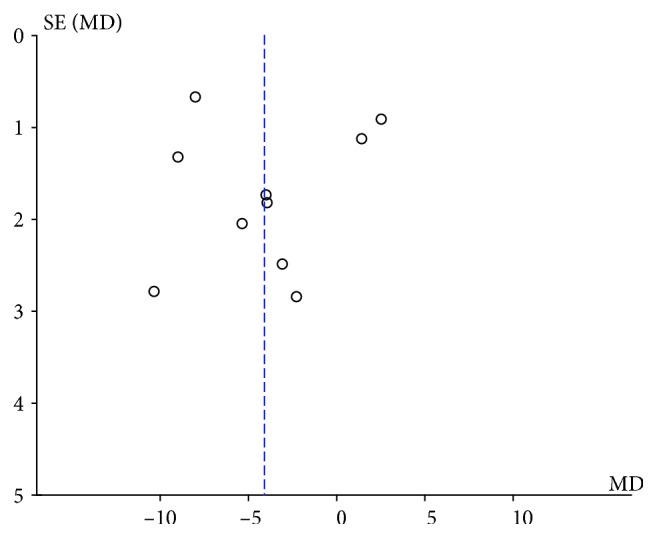
Funnel plot of included articles based on the data for FBG.

Substantial heterogeneity was observed in the meta-analyses for blood glucose and related indicators, triglycerides, and macrosomia. As the data on the duration and dose of probiotic intervention could not be grouped appropriately, subgroup analyses to investigate the potential source of heterogeneity were conducted according to the mean age of participants and the type of intervention only (Table 2). The results showed that the effects of probiotics on FBG, FSI, HOMA-IR, QUICKI, and macrosomia varied considerably by age, while none of the results of meta-analyses changed with the type of intervention. This indicates that the mean age of participants is a potential source of the substantial heterogeneity. Sensitivity analyses by excluding the studies with high risk bias showed that the results of meta-analyses were robust. The number of studies was smaller than 10 in all meta-analyses except the one for FBG. The funnel plot constructed based on the data for FBG was visually symmetric (Figure 7), providing no evidence for publication bias.

## 4. Discussion

This systematic review included 11 trials and assessed the effects of probiotic supplementation for 4-8 weeks on pregnancy outcomes, glycemic control, lipid profiles, and biomarkers of inflammation and oxidative stress in pregnant women with GDM. We identified three published systematic reviews that were similar to but actually different from the present one. Specifically, the systematic review by Taylor et al. [12] was interested in the effects of probiotics for treating GDM (i.e., conducted in women with GDM) but included only four trials. The systematic reviews by Peng et al. [38] and Zheng et al. [39] both included more than 10 trials, but among them, only four or five unique trials were conducted to assess the effects of probiotics for treating GDM (i.e., conducted in women with GDM), while the other trials assessed the preventive effects of probiotics (i.e., conducted in women without GDM) which is not relevant to the objective of the present systematic review. In addition, the three previous systematic reviews were mainly focused on glycemic control and lipid profiles. The main strength of the present review is that it included much more trials than previous ones, with most of the trials at a low risk of bias, and investigated both hard and surrogate outcomes.

Our meta-analyses showed that probiotic supplementation in women with GDM reduced the incidence of a newborn's hyperbilirubinemia and improved HDL-cholesterol and four biomarkers related to inflammation and oxidative stress, with no or low heterogeneity among studies. However, whether these results are sufficient to support the use of probiotics in the management of GDM remains uncertain, for two reasons. First, most of the above outcomes are surrogate markers rather than patient-important clinical endpoints. For example, inflammation and oxidative stress measures were investigated by some of the included studies because they were believed to be associated with cardiovascular disease [24, 32]. However, this argument is mainly based on low-level evidence from small cross-sectional or retrospective studies [40, 41]. Few prospective studies are available to corroborate the hypothesis that they are predictive of cardiovascular disease in later life in women with GDM. Even if the association does exist, the number of CVD events that are attributable to the change in those surrogate markers and the number of events that can be prevented by probiotics supplementation remain unknown. Thus, it may be hard to determine the clinical significance of the effects of probiotics based on currently available studies. Second, almost all studies included in the meta-analyses for the above outcomes (except one study for HDL-cholesterol) [35] were from Iran. It is unclear if there was any systematic difference between the Iranian studies and those from other countries. For example, in the meta-analyses on HDL-cholesterol (Figure 5(d)), the point effect estimates of Iranian studies all favored probiotics, whereas the effect estimate in Lindsay et al.'s study, which was from Ireland, favored the control group. If this was indeed a result from different countries of study (e.g., the study population or methodology was systematically different), the generalizability of our meta-analysis results would be undermined.

Our meta-analyses also found that probiotics could improve glycemic control and reduce triglyceride, but there was substantial heterogeneity among studies. Prespecified subgroup analyses according to age and intervention type showed that the heterogeneity was substantially reduced or even disappeared in a few subgroups, but persisted in most others (Table 2). A possible explanation for this is that the potential effect modification by the two subgroup factors could not be effectively investigated by our subgroup analyses, as the subgroups were formed based on aggregate data rather than individual data (which was not available to us) and they were not distinct from each other in terms of the status of the subgroup factor. For example, the subgroups with a mean age < 30 years actually had some participants older than 30 years, and similarly, those with a mean age ≥ 30 years had some participants younger than 30 years. This issue might lead to ecological bias and undermine the validity of subgroup comparison. Another possible explanation is that the substantial heterogeneity was mainly caused by other factors than age and intervention type, e.g., the species and dosage of probiotics. However, limited available data precluded us from doing further analyses on these factors. In addition, because of the surrogate nature of outcomes and the fact that the included studies are mostly from Iran, as mentioned above, the clinical significance and generalizability of the meta-analysis results on glycemic control and triglyceride are also uncertain.

In summary, the results of this systematic review should be interpreted with the following limitations taken into account: First, there was substantial heterogeneity in some of the meta-analyses, which was associated with but could not be completely attributable to the mean age of participants and intervention type. Other causes for the substantial heterogeneity remain to be investigated. Second, the fact that most studies came from Iran may undermine the generalizability of results to some extent. Third, potential publication bias could not be investigated effectively owing to the small number of studies included in most meta-analyses. Further studies conducted in different races of population, with larger sample size and focusing on hard outcomes rather than surrogate markers, are needed to validate the health effects of probiotics in women with GDM.

## 5. Conclusions

In conclusion, probiotic supplementation seemed to be able to reduce the risk of a newborn's hyperbilirubinemia and improve glycemic control, blood lipid profiles, and inflammation and oxidative stress in pregnant women with GDM. However, due to the heterogeneity among existing studies, the surrogate nature of outcomes, and/or the fact that most studies were from Iran, the clinical significance and generalizability of the above findings remain uncertain. Further studies are warranted to address the limitations of existing evidence and better inform the management of GDM.

## Figures and Tables

**Table 1:** Characteristics of the included studies.

Study	Year	Country	Intervention/control (sample size)	Age (intervention/control)	Duration (weeks)	Probiotic species	Total dose (CFU)
Ahmadi et al.	2016	Iran	Synbiotic/placebo (35/35)	28.5 ± 5.8/28.7 ± 3.4	6	Lactobacillus acidophilus Lactobacillus casei Bifidobacterium bifidum	6 × 10^9^
Babadi et al.	2018	Iran	Probiotic/placebo (24/24)	29.0 ± 4.2/28.8 ± 4.3	6	Lactobacillus acidophilus Lactobacillus casei Bifidobacterium bifidum Lactobacillus fermentum	8 × 10^9^
Badehnoosh et al.	2018	Iran	Probiotic/placebo (30/30)	27.8 ± 3.7/28.8 ± 5.4	6	Lactobacillus acidophilus Lactobacillus casei Bifidobacterium bifidum	6 × 10^9^
Dolatkhah et al.	2015	Turkey	Probiotic/placebo (29/27)	28.1 ± 6.2/26.5 ± 5.2	8	Lactobacillus acidophilus LA-5 Bifidobacterium BB-12 Streptococcus thermophilus STY-31 Lactobacillus delbrueckii bulgaricus LBY-27	>4 × 10^9^
Jafarnejad et al.	2016	Iran	Probiotic/placebo (41/41)	32.4 ± 3.1/31.9 ± 4.0	8	Streptococcus thermophilus Bifidobacterium breve Bifidobacterium longum Bifidobacterium infantis Lactobacillus acidophilus Lactobacillus plantarum Lactobacillus paracasei Lactobacillus delbrueckii subsp. Bulgaricus	15 × 10^9^
Kijmanawat et al.	2018	Thailand	Probiotic/placebo (28/29)	32.5 ± 5.0/30.7 ± 5.1	4	Bifidobacterium Lactobacillus	2 × 10^9^
Lindsay et al.	2015	Ireland	Probiotic/placebo (74/75)	33.5 ± 5.0/32.6 ± 4.5	6	Lactobacillus salivarius	1 × 10^9^
Nabhani et al.	2018	Iran	Synbiotic/placebo (45/45)	29.4 ± 5.8/30.3 ± 5.6	6	L. acidophilus L. plantarum L. fermentum L. gasseri	9.2 × 10^10^
Jamilian et al.	2018	Iran	Probiotic/placebo (29/28)	31.2 ± 5.9/29.9 ± 3.7	6	Lactobacillus acidophilus Bifidobacterium bifidum L. reuteri Lactobacillus fermentum	8 × 10^9^
Karamali et al.	2016	Iran	Probiotic/placebo (30/30)	31.8 ± 6.0/29.7 ± 4.0	6	Lactobacillus acidophilus L. casei Bifidobacterium bifidum	6 × 10^9^
Karamali et al.	2018	Iran	Synbiotic/placebo (30/30)	27.2 ± 5.9/26.2 ± 3.1	6	Lactobacillus acidophilus L. casei Bifidobacterium bifidum	6 × 10^9^

**Table 2:** The results of subgroup analysis according to intervention type and age.

Subgroup	Studies	Patients	Mean difference (95% CI) unless other specified	Heterogeneity (*I*^2^)%	*P* value
Macrosomia					
Intervention					
Probiotics	3	264	0.47 (0.10, 2.15)^∗^	56	0.47
Synbiotics	1	60	0.14 (0.01, 2.65)^∗^		
Mean age					
<30	2	120	0.13 (0.02, 1.08)^∗^	0	0.09
≥30	2	204	0.89 (0.45, 1.77)^∗^	64	
FBG					
Intervention					
Probiotics	8	510	-4.08 (-4.90, -3.26)	95	0.49
Synbiotics	2	160	-2.75 (-6.42, 0.92)	0	
Mean age					
<30	5	324	-6.84 (-7.96, -5.72)	63	<0.00001
≥30	5	346	-1.15 (-2.28, -0.01)	94	
FSI					
Intervention					
Probiotics	7	450	-2.24 (-3.28, -1.20)	81	0.54
Synbiotics	2	160	-3.90 (-9.07, 1.27)	57	
Mean age					
<30	4	264	-1.40 (-1.67, -1.13)	64	0.007
≥30	5	346	-2.41 (-3.10, -1.72)	80	
HOMA-IR					
Intervention					
Probiotics	7	450	-0.67 (-0.94, -0.40)	82	0.72
Synbiotics	3	160	-0.87 (-1.92, -0.19)	73	
Mean age					
<30	4	264	-0.42 (-0.49, -0.36)	59	0.01
≥30	5	346	-0.66 (-0.83, -0.49)	83	
QUICKI					
Intervention					
Probiotics	4	221	0.01 (0.01, 0.01)	74	0.86
Synbiotics	2	160	0.01 (0.01, 0.03)	60	
Mean age					
<30	4	264	0.01 (0.01, 0.01)	76	0.02
≥30	2	117	0.01 (0.01, 0.02)	0	
Triglycerides					
Intervention					
Probiotics	4	265	-17.51 (-27.47, -7.54)	6	0.71
Synbiotics	2	160	-20.71 (-34.62, -6.80)	86	
Mean age					
<30	3	208	-20.47 (-32.11, -8.82)	73	0.66
≥30	3	217	-16.84 (-28.12, -5.56)	36	

^∗^The effect measure is the risk ratio (95% CI). GDM: gestational diabetes mellitus; FBG: fasting blood glucose; FSI: fasting serum insulin; HOMA-IR: homeostasis model assessment insulin resistance; QUICKI: quantitative insulin sensitivity check index.
